# Infective endocarditis in dogs in the UK: 77 cases (2009‐2019)

**DOI:** 10.1111/jsap.13561

**Published:** 2022-11-06

**Authors:** M. Berrezaie, D. Connolly, J. Cruzado, E. Mederska, J. Dukes‐McEwan, K. Humm

**Affiliations:** ^1^ Department of Clinical Science and Services The Royal Veterinary College, Hawkshead Lane, Hatfield Hertfordshire AL9 7TA UK; ^2^ Southern Counties Veterinary Specialists, Unit 6, Forest Corner Farm, Hangersley, Ringwood Hampshire BH24 3JW UK; ^3^ Department of Small Animal Clinical Science, School of Veterinary Science, Leahurst Campus University of Liverpool, Chester High Road Neston CH64 7TE UK

## Abstract

**Objectives:**

To determine the causative organisms, clinical features and outcome of canine infective endocarditis in the UK.

**Materials and Methods:**

Medical records of three veterinary referral hospitals were searched for dogs with infective endocarditis between December 2009 and December 2019. Signalment, clinical signs, causative organism, valve affected, treatment and survival data were recorded.

**Results:**

Seventy‐seven cases with possible or definite infective endocarditis (according to the modified Duke criteria) were included. The majority were large breed (40/77 – 51.9%). There were 47 of 77 (61%) male dogs and the mean age was 7.3 ±3 years. A causative organism was identified in 26 of 77 (33.8%) cases. The most common organisms were *Escherichia coli* (7/27 – 25.9%), *Pasteurella* spp. (5/27 – 18.5%), *Staphylococcus* spp. (4/27 – 14.8%) and *Corynebacterium* spp. (4/27 – 14.8%). *Bartonella* spp. were not detected in any patients. The mitral valve was most commonly affected (48/77 – 62.3%). Clinical features were non‐specific, with lethargy being the most common clinical sign observed (53/77 – 68.8%). Fifty‐three dogs (68.8%) survived to discharge. The median survival time post discharge was 425 days (2 to 3650 days). The development of congestive heart failure was associated with a poorer outcome. Cardiac troponin concentration, antithrombotic use and the development of thromboembolism or arrhythmias were not significantly associated with outcome.

**Clinical Significance:**

Some dogs with infective endocarditis that survive to discharge can have a long lifespan. The inability to detect an underlying organism is common and *Bartonella* spp. may be a less prevalent cause of canine infective endocarditis in the UK than in the USA.

## INTRODUCTION

Infective endocarditis (IE) is a life‐threatening disease that is difficult to diagnose and manage in veterinary patients (Miller *et al*. 2004). It is caused by bacterial infection of the valvular endothelium and results in proliferative or erosive lesions leading to valvular insufficiency (Häggström 2010). The prevalence of IE varies between publications but is considered low in canine patients. An incidence of <1% has been reported in one veterinary hospital (MacDonald *et al*. 2004). Males appear to be at a greater risk of IE than females (Sisson & Thomas 1984, Miller *et al*. 2004). Previous studies have contradictory findings regarding breed predilections. Some suggest small breed dogs are more predisposed due to their predisposition to congenital heart defects, while others suggest medium to large breed dogs are over‐represented (Sisson & Thomas 1984, Miller *et al*. 2004, Romero‐Fernandez & Palermo 2019). Valvular endocardiosis appears to be a predisposing factor for IE in humans; however, this does not appear to be the case in dogs (Kiefer & Bashore 2012, Romero‐Fernandez & Palermo 2019).

IE can be difficult to diagnose ante‐mortem due to the non‐specific and variable clinical signs and limited diagnostic capabilities in general practice (Häggström 2010). However, a modified version of the human Duke criteria used for diagnosis of IE in dogs has been described (Sykes *et al*. 2006a). Although not part of the Duke criteria, cardiac troponin‐I (cTnI) is another supportive test for IE (Kilkenny *et al*. 2021). The most common bacteria identified in previous studies of dogs with IE were *Streptococcus* spp. and *Bartonella* spp. according to two case series in the USA (Sykes *et al*. 2006a, Reagan *et al*. 2022). Other commonly implicated organisms include *Staphylococcus* spp., *Escherichia coli*, *Pseudomonas* spp., *Erysipelothrix rhusiopathiae*, *Pasteurella* spp. and *Corynebacterium* spp. (Peddle & Sleeper 2007, Reagan *et al*. 2022). Previous studies indicate that *Streptococcus* spp. most commonly infects the mitral valve, *Bartonella* spp. tend to affect the aortic valve while *Staphylococcus* spp. display no valve predilection (MacDonald *et al*. 2004, Sykes *et al*. 2006a). Other Gram‐negative bacteria showed a predilection to infect the mitral valve (Sykes *et al*. 2006a). A bacteraemia is required for the development of IE; however, many cases have no clinically detectable source of infection, possibly because many dogs are already receiving antibiotic therapy before the start of a diagnostic work‐up (Romero‐Fernandez & Palermo 2019). The use of antithrombotics has been shown to increase survival time in IE patients (Reagan *et al*. 2022).

The prognosis for IE and its sequelae in dogs is guarded; in one retrospective case series, a survival rate of 50% was reported (Reagan *et al*. 2022). This appears to be dependent on the valve affected, however, with a shorter median survival time of just 3 days in dogs with aortic valve IE and of 476 days in dogs with mitral IE according to a previous study (MacDonald *et al*. 2004). The shorter survival time of aortic valve infections is thought to be due to its predisposition to *Bartonella* spp. colonisation (Sykes *et al*. 2006b). This can lead to aortic regurgitation which is less well tolerated than mitral regurgitation as it is associated with high afterload and possibly myocardial failure. A recent study has found a longer survival time of 71 days in dogs with aortic valve IE due to *Bartonella* spp. than previous studies (Reagan *et al*. 2022). Complications associated with IE include congestive heart failure (CHF), immune‐complex disease and thromboembolic disease (TED) which can manifest in many organs including the kidneys (Reagan *et al*. 2022). The development of CHF, TED and acute kidney injury (AKI) has been shown to be negatively correlated with survival (Reagan *et al*. 2022).

The scientific literature on canine IE is limited and focused on veterinary hospitals in the USA. The aim of this study was to address the gap in the literature on canine IE cases in the UK, specifically to describe signalment, presenting clinical signs, valve affected, causative bacterial species and outcome in these patients.

## MATERIALS AND METHODS

### Study design and inclusion criteria

This was a retrospective study and the medical records of three UK veterinary referral hospitals were searched for dogs diagnosed with IE between January 2009 and December 2019. Cases were classified as definite or possible IE based on the modified Duke criteria described by Keene (2002), Sykes *et al*. (2006a) and Ljungvall & Häggström (2017) (Table 1) or definite when the diagnosis was confirmed by *post‐mortem* examination. Cases were excluded if they had had previous cardiac surgery associated with the mitral valve repair programmes at two of the veterinary hospitals. Positive findings for IE on echocardiogram as described in Table 1 involved documenting changes in the normal heart anatomy such as thickening of the valves, vegetative lesions, which are often irregularly outlined and oscillatory (*i.e*. move independently from the valve) and associated valvular insufficiencies and/or elevated valve velocities (Ljungvall & Häggström 2017). The mitral valves were viewed from several angles to help distinguish between myxomatous nodular lesions (if degenerative valvular disease was present) and vegetations (Ljungvall & Häggström 2017). The echocardiograms were carried out by residents or board‐certified veterinary cardiologists at the veterinary hospitals. A simultaneously acquired single‐lead electrocardiogram (ECG) was reviewed during echocardiography, with a six or 12 lead ECG recorded according to clinical indication or clinician's preference. The presence (or absence) of arrhythmias was noted.

**Table 1:** The modified Duke criteria for the diagnosis of infective endocarditis in dogs

Major criteria	Minor criteria
Positive findings for endocarditis on echocardiogram, *e.g*. vegetative or erosive lesion, abscess	Pyrexia (≥39.3°C)
New valvular insufficiency: moderate to severe aortic insufficiency without subaortic stenosis (SAS)	New or worsening heart murmur
At least two separate microbial culture of blood (MCB) positive for a typical organism or three if common skin contaminant	Single positive MCB or serologic evidence of infection by indirect fluorescent antibody assay and/or by polymerase chain reaction
	Detection of vascular or embolic event, *e.g*. thromboembolism
	Immunologic event, *e.g*. immune‐mediated thrombocytopenia, glomerulonephritis, etc
	Prolonged intravenous (iv) catheterisation or infected iv catheterisation
	SAS
	Medium to large dog (>15 kg)
	History of steroid use with any of the above conditions

A definite diagnosis requires fulfilment of at least two major criteria or one major plus two minor criteria. A possible diagnosis requires fulfilment of one major and at least one minor criteria or three minor criteria (adapted from Keene 2002, Sykes *et al*. 2006a and Ljungvall & Häggström 2017)

### Medical record search

Electronic medical records from each referral hospitals were searched for dogs diagnosed with endocarditis between 2009 and 2019. The software used included VetCompass, Tristan and Rx‐Works. Medical records were searched using the keywords “endocarditis” in three centres and “new heart murmur,” “pyrexia” and “lethargy” in one centre. The medical records were searched by two operators in one centre and one operator each in both other centres. The dates the medical records were searched were May and July 2021.

### Data extracted from records

Clinical features were recorded including patient signalment, presenting clinical signs, Duke criteria fulfilment, microbial culture of blood (MCB) and *Bartonella* spp. polymerase chain reaction (PCR), valves involved, circulating cTnI concentrations, antibiotic therapy (before and post IE diagnosis), other therapy started post IE diagnosis, any comorbidities, hospitalisation length, complications (development of CHF, TED, arrhythmias and renal complications) and patient outcome. To evaluate outcome for dogs that survived hospital discharge, the primary veterinary practices of patients were contacted to determine whether they were known to be alive, or if they had died or were euthanased. The date of their euthanasia or natural death was gathered to the nearest month. If a dog died and a *post‐mortem* consent was provided, *post‐mortem* examination was carried out. Bacterial culture samples were taken aseptically from cardiac tissue. Ethical approval was gained to contact the veterinary practices from all institutions (ethical review reference number SR2020‐0120; VREC1092). The ethical approval SR2020‐0120 covers two centres. The development of complications was recorded as follows; CHF was diagnosed either on *post‐mortem* by board‐certified clinical pathologists or by findings of cardiogenic pulmonary oedema (*e.g*. enlarged cardiac silhouette, enlarged pulmonary vasculature and infiltrative pulmonary patterns) by thoracic radiographs identified by board‐certified veterinary radiologists. Thromboembolic events were defined as visualisation of infarcts or thrombus either at *post‐mortem* examination or by abdominal ultrasound or computed‐tomography by board‐certified clinical pathologists or radiologists respectively. Renal complications were defined as cases with serum creatinine concentrations above the normal reference range in animals with concurrent isosthenuria or hyposthenuria and no history of chronic kidney disease.

### Bartonella detection

Detection of *Bartonella* spp. for this study was carried out by DNA extraction and qPCR for centre A, PCR alone for centres B and C.

### Collection of blood cultures

In centre A, aseptic collection of three, 3 to 10 ml aliquots were collected from three different veins following sterile preparation, all taken at the same time. The whole blood was then subcultured onto blood agar and MacConkey agar for aerobic and anaerobic cultures and incubated at 37°C for 7 days. In centre B, aseptic collection of three, 5 ml aliquots were collected from three different veins following sterile preparation in a time frame of 60 minutes. The whole blood was then subcultured onto blood agar for aerobic and anaerobic cultures and incubated at 37°C for 7 days. In centre C, aseptic collection of three, 2 to 5 ml aliquots were collected from three different veins following sterile preparation, 30 minutes apart. The whole blood was then subcultured onto Signal Blood Culture System (ThermoFisher) for aerobic and anaerobic cultures and incubated at 38°C for 4 to 7 days.

### Statistical analysis

Continuous variables were assessed for normality using the Shapiro–Wilk test. Normally distributed data were reported as mean ±sd and non‐normally distributed data as median (minimum to maximum range). Data were analysed using the statistical analysis programme GraphPad Prism Version 9.0 (GraphPad Software). Definite and possible cases of endocarditis were analysed together. Kaplan–Meier survival curves were constructed and the log‐rank test was used to compare the following populations: dogs with different valve infections, use of antithrombotics, development of CHF, TED and arrhythmias. Survivors were censored on the last day of follow‐up. Cases lost to follow‐up before 1 month after discharge were excluded from the patient outcome analysis. Values of P < 0.05 were set as significant.

## RESULTS

The medical record search identified 287 patients at referral centre A, 49 records at centre B and 42 records at centre C that were eligible for assessment. In total, 77 cases were eligible for the study, the rest were excluded as the final diagnosis was not IE and they did not fulfil enough criteria to be defined as possible or definite cases of IE.

### Signalment

A total of 77 cases were included in this study. There were 37 cases from centre A, nine cases from centre B and 31 cases from centre C. There were more male (neutered: n=33/77, 43%; entire: n=14/77, 18%) than female (neutered: n=21/77, 27%; entire: n=9/77, 12%) dogs. The mean age of all dogs was 7.3 ±3 years. The most common breeds were Labrador retrievers and their crosses (n=15/77, 19%), followed by Border collies (n=9/77, 12%) and boxers and their crosses (n=8/77, 10%). There were more large breed dogs (>25 kg) (n=40/77, 52%) than medium breed dogs (10 to 25 kg) (n=28/77, 36%) and small breed dogs (<10 kg) (n=9/77, 12%).

### Common clinical signs

The median duration of illness before admission was 7 days (0 to 334 days). The most common clinical signs on admission are summarised in Table 2. Other clinical signs recorded were blindness, ptyalism and epistaxis in one dog each. Seventy‐one (92%) dogs presented with multiple clinical signs.

**Table 2:** Summary of the main clinical signs observed in this study

Clinical signs	Number of cases
Lethargy/weakness	53
Pyrexia (≥39.3°C)	47
Locomotor problems, *e.g*. shifting, acute or chronic lameness, joint pain or effusions	34
Neurological signs, *e.g*. obtundation, head tilt, nystagmus	16
Collapse	13
Diarrhoea	11
Weight loss	8
Vomiting	7
Polydipsic/polyuric	7
Cough	5
Oculonasal discharge	4
Abdominal pain	2

### Comorbidities

Thirty‐five (45%) of 77 patients had no co‐morbidities reported before the development of IE. Of the 42 remaining cases, the most common comorbidities were osteoarthritis (n=8, 19%), skin disorders (aural infection, cellulitis, grass seed foreign body and associated infection, aural haematoma and wounds) (n=7, 17%), dental disease (n=6, 14%), urinary tract infections (n=5, 12%), discospondylitis (n=3, 7%), prostatitis (n=2, 5%), gastroenteritis (n=2, 5%), septic peritonitis (n=2, 5%), closed pyometra (n=1, 2%) and bronchopneumonia (n=1, 2%). Other comorbidities included neoplasia (n=4, 10%), myxomatous mitral valve disease (MMVD) (n=2, 5%), keratoconjunctivitis sicca (n=1, 2%), epilepsy (n=1, 2%), conjunctivitis (n=1, 2%) and meningitis of unknown aetiology (n=1, 2%). Some dogs had multiple comorbidities (n=4).

### Duke criteria fulfilment

**Fig 1:**
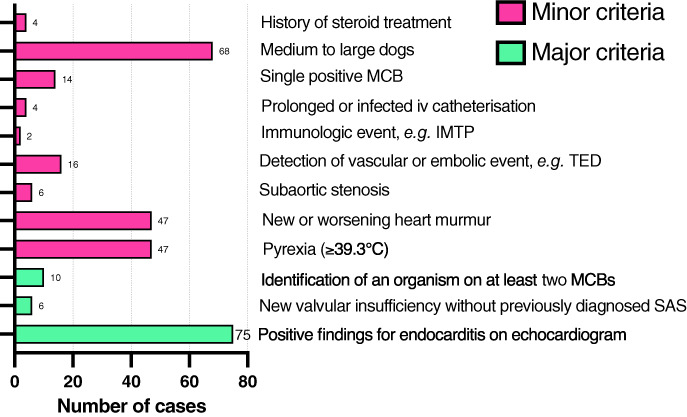
Histogram showing the number of cases which fulfilled each of the modified Duke's criteria. MCB Microbial culture of blood, iv Intravenous, IMTP Immune‐mediated thrombocytopaenia, TED Thromboembolism, SAS Sub‐aortic stenosis

Out of 77, 67 (87%) dogs were classified as definite endocarditis, and 10 (13%) as possible according to the modified Duke criteria. Exclusion of possible cases did not impact the results or statistical analyses. Figure 1 summarises how many cases fulfilled each of the modified Duke's criteria.

Three dogs had prolonged intravenous (iv) catheterisation sites and one had an infected iv catheter site. One of the dogs with the prolonged catheterisation sites had a vascular access port placed. This dog subsequently developed a tricuspid IE. The dog with the infected catheter site developed aortic IE and the other two dogs developed mitral IE.

Ten dogs were submitted for *post‐mortem* at which point a definite diagnosis of endocarditis was confirmed by bacterial culture of cardiac tissue and characteristic valve pathology. Microbiology laboratory reports of these samples did not interpret any of the cultures to be potentially contaminated.

Eight of the 10 dogs were already diagnosed as definite endocarditis cases before *post‐mortem* examination. However, the remaining two cases were initially classed as “possible” endocarditis cases before the *post‐mortem* examination. One of these dogs did not have an MCB submitted ante‐mortem and another had a negative MCB result. Following confirmation of the *post‐mortem* results, these two dogs were then classified as “definite” endocarditis. In four cases, the records did not state how many MCBs were collected; these were classed as a single positive MCB.

### Infecting organism and valve involvement

**Fig 2:**
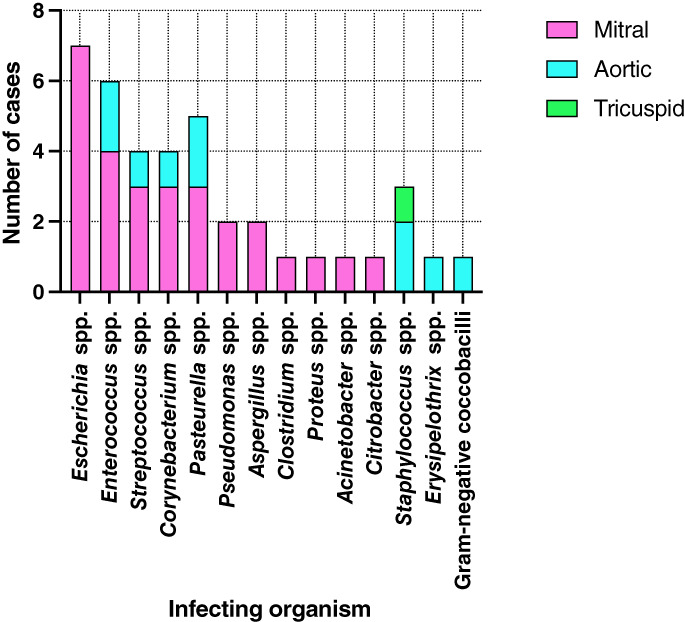
Histogram showing the infecting organisms and cardiac valves involved in the 26 cases with positive blood culture

A causative organism was identified in 26 of the 77 cases (34%). Seven dogs had multiple organisms grown on MCB (in four cases this was detected on *post‐mortem* examination). Figure 2 shows the distribution of organisms confirmed by blood culture and which valve they infected. There was a negative blood culture or blood cultures in 36 (59%) of 77 dogs, with this being more common in referral centre B (n=7/8, 87.5%) compared to referral centre A (n=13/32, 37.5%) and referral centre C (n=16/22, 72.7%). One dog had no blood culture taken but IE was confirmed by *post‐mortem* examination. No blood culture or *post‐mortem* examination was performed in 14 (18.2%) of the 77 cases. Six of these cases died or were euthanased within 3 days of admission (range 1 to 5 days). Two of these 14 dogs were classified as possible endocarditis, the other 12 were classified as definite endocarditis. Table 3 shows how the 12 cases that were classified as definite endocarditis fulfilled this classification despite not having a blood culture or *post‐mortem* carried out. A PCR test for *Bartonella* spp. was performed for 13 dogs, the results were negative for all 13.

**Table 3:** Summarising which criteria were fulfilled in the 12 cases with no blood cultures or no *post‐mortem* examinations performed

Minor criteria								
Case	Positive findings for endocarditis on echocardiogram	Over 15 kg	Pyrexia	New onset heart murmur	Infected iv site	Detection of TE	Detection of IMTP	Subaortic stenosis
1	Yes	Yes	Yes	Yes	Yes			
2	Yes		Yes	Yes		Yes	Yes	
3	Yes	Yes	Yes					
4	Yes		Yes	Yes				
5	Yes	Yes		Yes				
6	Yes	Yes		Yes				Yes
7	Yes	Yes		Yes				
8	Yes	Yes	Yes					
9	Yes	Yes	Yes					
10	Yes	Yes	Yes	Yes				
11	Yes	Yes	Yes					
12	Yes		Yes	Yes				

Out of the 77 cases, the mitral valve was infected in 48 cases (62.3%), the aortic in 18 cases (23.7%) and the tricuspid in two cases (2.6%). The aortic and mitral were both infected in six cases (7.9%), while the aortic and tricuspid, and the aortic, tricuspid and pulmonic were infected in one case each (1.3%). One dog did not have an echocardiogram done. This dog was classified as possible endocarditis as it did not fulfil any of the major criteria and had four minor criteria. Six dogs had mural lesions in addition to a valve lesion (8%): two dogs with aortic IE had a lesion on the right interatrial septum, one dog with aortic IE had a lesion extending into the right atrium, one dog with aortic and tricuspid IE had a lesion extending into the myocardium of the atrioventricular region, one dog with mitral IE had an lesion extending into the myocardium of the left ventricle and one dog with mitral IE had a lesion in the ventricular apical lumen and another lesion extending into the left ventricular outflow tract.

### Hospitalisation and patient outcome

**Fig 3:**
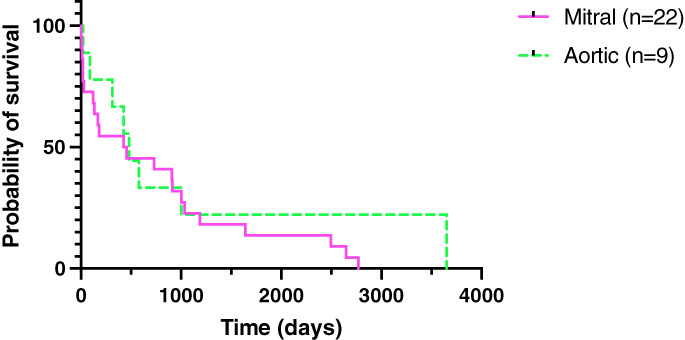
Kaplan–Meier survival curves of 31 dogs that were discharged from each referral centre with previously diagnosed aortic and mitral valve infective endocarditis

Out of the 77 patients, 19 dogs were euthanased and five died spontaneously at the hospital (30%). Of the 19 that were euthanased, 13 (68%) had mitral IE, four (21%) had aortic IE, one (5%) had tricuspid IE and one had mitral and aortic valve IE (5%). Of the five that died, three (60%) had mitral IE and two (40%) had aortic IE. The median hospitalisation length of cases that died or were euthanased was 2 days (0 to 10 days). Of the 77 cases, 53 (69%) survived to discharge with a mean length of hospitalisation of 7.1 days ±3.9 days. Dogs that were discharged from the hospitals with aortic valve endocarditis lived a median of 480 days (range 22 to 3650 days) while dogs with mitral valve endocarditis lived a median of 440 days (range 2 to 2769 days) (Fig 3); survival times were not significantly different for the site of endocarditis. Fifteen dogs (28%) were lost to follow‐up and excluded from this analysis. The dog with tricuspid valve endocarditis lived 152 days. Dogs with both aortic and mitral valve endocarditis lived a median of 121 days (2 to 1065 days) and the dog with aortic, tricuspid and pulmonic valve endocarditis lived for 1825 days.

The median hospitalisation time of dogs with mitral valve IE was 5 days (n=48, 0 to 15 days), for aortic valve IE it was 4.5 days (n=18, 1 to 11 days), and for tricuspid valve IE it was 4.5 days (n=2, 0 to 9 days). The mean hospitalisation length of dogs with mitral and aortic valve IE was 7 days (n=5, ±4.5 days). The hospitalisation time of the dog with aortic and tricuspid valve IE was 8 days and the dog with aortic, tricuspid and pulmonic valve IE was 5 days. The mean hospitalisation time of dogs with single valve IE was 5.7 days (n=68, ±3.8 days) and that of multiple valve IE was 6.9 days (n=7, ±3.8 days).

### Antimicrobial therapy

Of the 77 cases, 52 (68%) had received either injectable or oral antimicrobial therapy when they presented at the referral hospitals, as summarised in Table 4. The median time antimicrobial therapy was prescribed by the referring veterinary practice was 7 days before referral to the hospitals (1 to 56 days). Topical antibiotics were excluded from this analysis.

**Table 4:** Summary of systemic antibiotic therapy received before admission at the referral hospitals

Antibiotic	Number of cases
Amoxicillin–clavulanic acid	18
Amoxicillin–clavulanic acid, fluoroquinolone (enrofloxacin, marbofloxacin)	6
Amoxicillin–clavulanic acid, metronidazole, fluoroquinolone (enrofloxacin, marbofloxacin)	5
Fluoroquinolones (enrofloxacin, marbofloxacin, pradofloxacin)	4
Metronidazole	3
Amoxicillin–clavulanic acid, metronidazole	3
Penicillins (amoxicillin, amoxicillin–clavulanic acid), clindamycin	2
Tetracyclines (doxycycline, oxytetracycline)	2
Cefalexin, marbofloxacin	1
Cefalexin, metronidazole	1
Enrofloxacin, metronidazole	1

Of the 52 dogs that received antimicrobial therapy before referral, 24 subsequently showed negative MCB at the referral hospitals (46%). Of the remaining 25 dogs that had not received antibiotics before referral, 12 had negative MCB (48%).

The most common antibiotic therapies prescribed at the referral centres were amoxicillin–clavulanic acid (Synulox; Zoetis) (Augmentin; GSK) (Co‐amoxiclav; Sandoz limited) and a fluoroquinolone (Baytril; Elanco) (Marbocyl; Vetoquinol) (Marfloquin; Virbac) (n=15, 22%) amoxicillin–clavulanic acid and a fluoroquinolone with metronidazole (Metrobactin; Dechra) (Metronidazole; Braun) (n=10, 15%), fluoroquinolone, cephalosporin (Zinacef; GSK) (Therios; Ceva), (Convenia; Zoetis) (Rilexine; Virbac) and metronidazole (n=4, 6%). These were administered either iv, subcutaneously or by mouth. Nine (12%) of the 77 dogs did not receive antibiotic treatment at the hospital as they either died or were euthanased before therapy was started.

Of the 53 dogs that survived to discharge, the most common antibiotic protocol prescribed once discharged was 2 to 12 weeks of amoxicillin–clavulanic acid and a fluoroquinolone (n=23, 43%) by mouth. Four dogs had markedly prolonged therapy for periods of 5 to 22 months. Amoxicillin–clavulanic acid, enrofloxacin and metronidazole was used in nine dogs (17%).

### Other therapies

**Fig 4:**
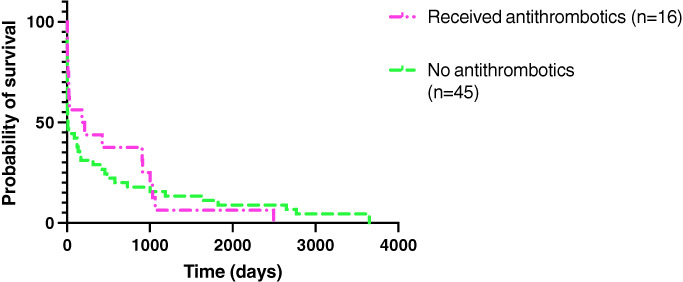
Kaplan–Meier survival curves comparing dogs that received antithrombotic medications and those that did not

A list of other therapies initiated on the diagnosis of IE is summarised in Table 5. Other therapies used included metoclopramide (Emeprid; CEVA), mexiletine (Mexiletine HCl; Summit) and amiodarone (Amiodrone; Covetrus) in one case each. Therapies intended solely for analgesia were excluded from this analysis, *e.g*. non‐steroidal anti‐inflammatory drugs, opioids etc. Antithrombotic medication (clopidogrel and/or aspirin) was used in 18 (23%) of 77 dogs, four of which developed TED. Figure 4 summarises the survival curves between dogs that received antithrombotic medication and those that did not. Sixteen dogs were excluded from this graph as they were lost to follow‐up. The log‐rank test showed no significant difference in the survival time between these groups.

**Table 5:** Summary of medication other than antimicrobials administered to patients diagnosed with infective endocarditis at the referral hospitals

Medication	Number of cases
Clopidogrel	14
Maropitant	10
Omeprazole	9
Furosemide	8
Pimobendan	8
Benazepril	7
Aspirin	6
Ranitidine	5
Sucralfate	4
Beta blocker (sotalol, atenolol)	4
Prednisolone	3
Amlodipine	3
Anticoagulant (dalteparin)	3
Mirtazapine	2

Name of drug manufacturers: Clopidogrel (Clopidogrel; Milpharm Limited) (Clopidogrel; Summit); Maropitant (Prevomax; Dechra) (Cerenia; Zoetis); Omeprazole (Omeprazole; Mylan) (Omeprazole; Bowmed Ibisqus Limited); Furosemide (Dimazon; MSD Animal Health) (Furosemide; Millpledge Veterinary); Pimobendan (Vetmedin; Boehringer Ingelheim); Benazepril hydrochloride (Fortekor; Elanco); Aspirin (Aspirin; Almus); Ranitidine (Zantac; GlaxoSmithKline) (Ranitidine; Summit); Sucralfate (Sucralfate; Summit); Solatol hydrochloride (Solatol hydrochloride; Tillomed Laboratories Ltd) (Sotalol; Almus); Atenolol (Atenolol; Summit) (Atenolol; Crescent); Prednislone (Prednicare; Animalcare Limited); Amlodipine (Istin; Pfizer) (Amlodip; CEVA); Dalteparin (Fragmin; Pfizer); Mirtazapine (Mirtazapine; Summit) (Mirtazapine; Milpharm Limited)

### Complications of IE

**Fig 5:**
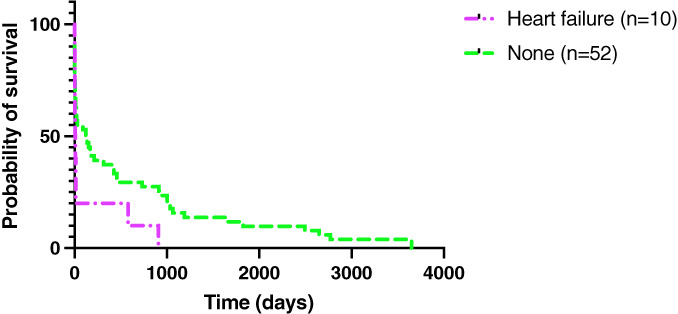
Kaplan–Meier survival curves comparing dogs that developed congestive heart failure and those that did not

Eleven (14%) out of 77 dogs developed CHF. These included nine (82%) dogs that developed left‐sided CHF, five (56%) of which were mitral valve IE, two (22%) had mitral and aortic valve IE and two (22%) had aortic valve IE. One (9%) dog developed biventricular CHF with an aortic valve IE and one (9%) dog developed mitral valve IE but the *post‐mortem* analysis did not specify what side CHF the dog developed. The median survival time of dogs that developed CHF with IE was 5 days (range 0 to 908 days). Figure 5 summarises the survival curves between dogs that developed CHF and those that did not. Fifteen dogs were excluded from this analysis as they were lost to follow‐up. The log‐rank test showed a significant difference between the survival time of these two groups (P=0.0440).

**Fig 6:**
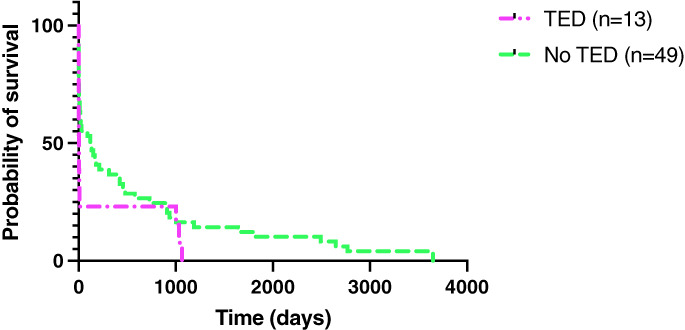
Kaplan–Meier survival curves comparing dogs that developed thromboembolic events and those that did not

Sixteen (21%) out of 77 dogs developed TED. Eight (50%) dogs had renal TED, seven (43%) dogs had splenic TED, four (25%) dogs had TED in their musculature, two (13%) dogs had liver TED and one (6%) dog had an aortic TED. Some dogs developed TED in multiple locations. Figure 6 summarises the survival curves of dogs that developed TED and those that did not. Fifteen dogs were excluded from this analysis as they were lost to follow‐up. The log‐rank test showed no significant difference between the survival time of dogs that developed TED and those that did not.

**Fig 7:**
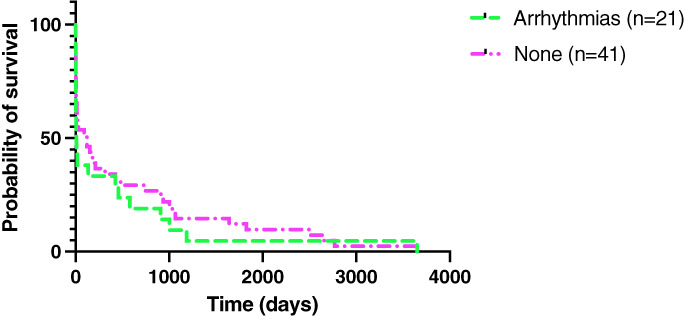
Kaplan–Meier survival curves comparing dogs that developed arrhythmias and those that did not

Twenty‐seven (35%) out of 77 dogs developed arrhythmias. Ventricular arrhythmias recorded included ventricular premature complexes (n=14, 52%), accelerated idioventricular rhythm (n=12, 44%), ventricular tachycardia (n=4, 15%) and ventricular bigeminy or trigeminy (n=2, 7%). Atrial arrhythmias recorded included supraventricular tachycardia (n=4, 15%) and supraventricular premature complexes (n=2, 7%). Four dogs had atrioventricular block, which was characterised as first degree in three dogs, second degree in one dog and third degree in one dog. Some dogs showed multiple types of arrhythmias. Figure 7 summarises the survival curves of dogs that developed arrhythmias and those that did not. Fifteen dogs were excluded from this analysis as they were lost to follow‐up. The log‐rank test showed no significant difference between the survival times of dogs that developed arrhythmias and those that did not.

AKI was observed in four (5%) of the 77 cases and the median survival time of these cases was 2 days (range 0 to 908).

One dog was diagnosed with a tract connecting the left ventricle and right atrium (Gerbode defect), presumed as a complication of IE. This was diagnosed on echocardiographic examination and confirmed at *post‐mortem*. This dog had aortic valve IE and had been diagnosed with congenital SAS. The dog subsequently developed third degree AV block and was euthanased on the second day of hospitalisation due to clinical worsening.

### Cardiac troponin level measurement

Table 6 summarises the cTnI concentrations in the 30 dogs in which measurements were taken.

**Table 6:** Summary of cTnI levels

	Median cTnI level (ng/ml) (range) (reference, <0.23 ng/ml)
All dogs (n=30)	2.9 (0.104 to 180)
Dogs that survived until discharge (n=25)	2.9 (0.104 to 180)
Dogs that died or were euthanased (n=5)	6.3 (1.05 to 27.5)

## DISCUSSION

This multi‐centre study represents the first review of canine IE in a referral population of dogs in the UK and the second largest case series of IE to date. Large and medium breed dogs appear to be more predisposed to developing IE than small breed dogs, as has been described in previous veterinary studies (Sisson & Thomas 1984, Peddle & Sleeper 2007, Kilkenny *et al*. 2021, Reagan *et al*. 2022). However, reasons for this remain unclear. The mean age at which dogs were infected with IE in this study was similar in males and in females. A higher proportion of middle‐aged to older dogs were reported with IE in this study as noted in previous studies (Sisson & Thomas 1984, Sykes *et al*. 2006b, Kilkenny *et al*. 2021, Reagan *et al*. 2022). This may be due to age‐related senescence of the immune system, which has been shown to increase the incidence of infection in older pets (Day 2010). Similar to previous studies, we show that male dogs have a greater predisposition to developing IE than female dogs (Sisson & Thomas 1984, MacDonald *et al*. 2004, Reagan *et al*. 2022). Studies have shown sex differences in immune components with female dogs displaying stronger cell‐mediated and humoral responses, greater numbers of CD8 T‐cells and higher immunoglobulin levels than males which may account for this difference (Blount *et al*. 2005, Sundburg *et al*. 2016).

The most common organisms that were cultured in this study were *E. coli*, *Staphylococcus* spp. and *Pasteurella* spp., which have all been reported in previous USA studies (MacDonald *et al*. 2004, Sykes *et al*. 2006a, Reagan *et al*. 2022). The mitral valve was most commonly infected in this study as shown in recent studies (Kilkenny *et al*. 2021; Reagan *et al*. 2022). This differs from previous studies however where both the aortic and mitral valve were frequently affected (Sykes *et al*. 2006a). One of the reasons for this is likely linked to the lack of *Bartonella* spp. IE cases, which appear to preferentially affect the aortic valve (MacDonald *et al*. 2004). However, it is also possible that *Bartonella* infections were missed due to a low level of PCR testing (in only 17% of cases), particularly given the high level of cases where no causative organism was detected (66%). A recent study found a 3% seroprevalence of *Bartonella* spp. in UK dogs (Alvarez‐Fernandez *et al*. 2018). A similar seroprevalence was found in USA dogs at 3.6%; however, this increased to 36 and 52% when dogs were co‐exposed to *Ehrlichia canis* or *Babesia canis*, respectively (Alvarez‐Fernandez *et al*. 2018). Neither *E. canis* or *B. canis* are thought to be endemic in the UK, which may explain why *Bartonella* spp. were not detected in our patients (Bird 2016, Wright 2018). Research shows that PCR testing is no more sensitive at detecting *Bartonella* spp. than blood cultures (Meurs *et al*. 2011, Roura *et al*. 2018), however, this depends on what samples were used to run the PCRs and how the blood samples were cultured. Studies have shown that using only valve tissue samples rather than blood samples and a pre‐enrichment culture before PCR testing may increase *Bartonella* positive results (MacDonald *et al*. 2004, Davis *et al*. 2020). A recent study utilised serology, PCR and blood cultures to aid their identification of *Bartonella* spp. as a cause of IE (Reagan *et al*. 2022). These techniques were not utilised in this study. Thus, performing both MCB, serology and PCR simultaneously may improve the detection of *Bartonella* spp. in IE patients, and may be required to prove that *Bartonella* spp. is not a major cause of IE in the UK (Meurs *et al*. 2011).

Nearly, half the MCBs in this study were negative and this was not related to antimicrobial therapy before referral. This was shown by the lack of differences in the number of MCBs between groups that did and did not receive antimicrobials before referral. This may be due to the ability of some bacteria to invade macrophages and reside in cells as quiescent intracellular reservoirs, which may help protect it against the immune system and antimicrobial therapy (Croxen & Finlay 2009). Other common reasons for obtaining negative MCBs include infections by non‐bacterial organisms such as *Aspergillus* spp. or fastidious organisms such as *Chlamydia* spp. or *Mycoplasma* spp. which have been shown to cause endocarditis in humans (Sykes *et al*. 2006a, Habib *et al*. 2010). *Aspergillus* spp. were cultured in two dogs in this study; unfortunately, its diagnosis can be missed as it is a slow‐growing organism and therefore takes longer to isolate from MCBs (Pasha *et al*. 2016). Fungal endocarditis lesions have been shown to embolize easily in humans and should therefore be suspected in patients with negative MCBs and signs of embolic disease.

Successful treatment of IE is based on early diagnosis and immediate, aggressive treatment to minimise secondary complications. Selection of the appropriate treatment is based on culture and sensitivity testing; however, while the culture results are pending, empirical treatment with a broad‐spectrum antibiotic such as an aminoglycoside, beta‐lactam or fluoroquinolone is recommended (Häggström 2010). The most common antibiotic therapy protocol used in this study (amoxicillin–clavulanate and enrofloxacin in 43% of patients) was similar to that proposed in previous literature (MacDonald 2010). Although current expert opinion suggests 4 to 6 weeks of antibiotic therapy (Häggström 2010), some patients in this study received much longer courses. Such long courses need to be carefully considered and patients monitored closely to determine if antibiotic therapy is still required as poor antimicrobial stewardship increases the risk of antimicrobial resistance (Schuts *et al*. 2016). Current guidelines in human cases of IE also suggest 4 to 6 weeks of antibiotic therapy, and longer courses are only indicated in cases of prosthetic valve IE (Baddour *et al*. 2015).

The comorbidities in dogs with endocarditis noted in this study are similar to those previously described (Sykes *et al*. 2006a, MacDonald 2010). The most common comorbidities were a history of osteoarthritis, skin infections and periodontal disease.

Although osteoarthritis is unlikely to be related to the development of IE in dogs, it can be a precursor to immune‐mediated polyarthritis or septic arthritis when combined with a generalised infection and any lameness or joint effusions should be investigated (MacDonald 2010). Skin abscesses and wounds have also been shown as portals of entry in a previous study (Sykes *et al*. 2006a). A link between endocarditis and periodontal disease has been shown in dogs (Pereira dos Santos *et al*. 2019). One study suggests that chronic inflammation of the oral cavity in the presence of bacterial flora may lead to endocarditis due to the development of high bacteraemia particularly in dogs with stage 3 periodontal disease (Glickman *et al*. 2009). However, other studies challenge this association (Sykes *et al*. 2006a, Peddle *et al*. 2009). Unfortunately, the stage of dental disease was not recorded in the patients in this study. The canine oral microbiome has been shown to be highly diverse and up to 38.2% of species are unculturable, thus these may also account for some of our negative MCB (Riggio *et al*. 2011). Although only six of our patients presented with a history of periodontal disease, up to 64.5% of dogs are affected with the disease in the general population (Robinson *et al*. 2016) and so it is likely that this was under‐reported in the patient records. In addition, the incidence and severity of periodontal disease increases with age which correlates with the higher number of middle age to older dogs affected by endocarditis as seen in this study (Wallis *et al*. 2019). From our data, endocarditis seems a rare sequela of periodontal disease.

Congenital and acquired cardiac diseases were previously shown to predispose dogs to endocarditis (Romero‐Fernandez & Palermo 2019). Only two dogs had acquired cardiac disease (MMVD), thus this was not considered to be a major predisposition to IE in this referral population. Six dogs had underlying congenital heart disease (SAS) which has been previously suggested to predispose dogs to IE due to creating turbulent blood flow and damage to the aortic cusps (MacDonald 2010). In addition, SAS is one of the most common congenital heart conditions in large breed dogs which may account for their predisposition to IE (Ontiveros & Stern 2021). Male dogs have also been shown to be predisposed to SAS which may also partly explain their higher prevalence (Schrope 2015). A recent study did not diagnose any congenital SAS in their IE cases thus further studies are indicated to investigate this link (Reagan *et al*. 2022). One dog showed a Gerbode type defect which is thought to be secondary to destruction of the interventricular septum by bacterial IE (Peddle *et al*. 2008).

Contrary to previous studies (Sykes *et al*. 2006b, Reagan *et al*. 2022), the development of TED and the use of antithrombotics were not shown to have a significant effect on survival. However, it appears logical that the use of antithrombotics would be beneficial in helping to decrease the size of vegetative lesions as research has shown that lesions may shelter bacteria from the immune system (Liesenborghs *et al*. 2020). To confirm these results, studies with larger sample size are necessary.

The development of arrhythmias was not shown to have an effect on survival in this study, as previously shown (Sykes *et al*. 2006b). It is possible that in the majority of cases that developed arrhythmias they were not severe or prolonged enough to affect survival. Previous studies indicated that the development of AKI was associated with mortality; however, too few dogs developed AKI in this study to allow analysis (Sykes *et al*. 2006b; Reagan *et al*. 2022). In fact, in agreement with Reagan *et al*., CHF was the only complication of IE that was found to have a significant effect on survival in this study (Reagan *et al*. 2022).

The most common clinical signs were non‐specific and similar to those described in the literature (Peddle & Sleeper 2007). Interestingly, a new or worsening heart murmur was only diagnosed in 47 of the 77 patients. In some cases, dogs had a pre‐existing heart murmur and therefore did not meet this criterion. As this is a minor criterion in the modified Duke criteria, it is essential that the lack of a new or worsening heart murmur on initial examination does not rule out endocarditis as a differential diagnosis in a septic patient. Although only 30 of our patients had serum cTnI levels measured, it was not shown to be helpful as a prognostic indicator. A recent study has shown that serum cTnI concentrations above >0.625 ng/ml are supportive of a diagnosis of IE (Kilkenny *et al*. 2021). This cut‐off could be useful as an additional minor criterion in the Duke's modified criteria; however, it has a high specificity and a low sensitivity, therefore, it must be used within the context of the overall clinical picture.

The survival to discharge of dogs with IE in this study was found to be better than in older USA studies, 68% compared to 22 and 56% previously reported (MacDonald *et al*. 2004, Sykes *et al*. 2006b). This correlates with a more recent USA study on IE which also found a higher survival to discharge (70%) (Reagan *et al*. 2022). Interestingly, there was no significant difference between the survival times of dogs with mitral and aortic IE compared to previous USA studies which reported mitral valve infections to have the longest survival time and aortic valve infections to have the shortest survival time (MacDonald *et al*. 2004). These differences may be linked to the lack of *Bartonella* spp. detected in our patients as infection with this bacterium has been shown to be negatively correlated with survival and preferentially infects the aortic valve (Sykes *et al*. 2006a). Further studies with larger sample numbers may help validate these findings in both the UK and the USA. Interestingly, dogs with both mitral and aortic valve IE had the shortest survival times as in a previous study and likely represent advanced disease leading to degenerative structural changes in the heart and therefore a worsened prognosis (Reagan *et al*. 2022). Of the dogs that developed a tricuspid and/or pulmonic valve IE, only one tricuspid valve IE case had a history of having a jugular vascular access port placed. This likely would have been the portal of entry of the infection. Tricuspid and pulmonic valves are rarely affected by IE due to the higher pressures sustained on the left‐sided valves which predisposes the mitral and aortic valve to endothelial damage (Frontera & Gradon 2000). It is thought the relatively higher oxygen concentration of the left‐sided circulation is also more supportive of bacterial growth (Frontera & Gradon 2000).

There are a number of limitations in this study, many common to retrospective studies relying on data retrieval. One limitation is that there were multiple different operators who carried out the echocardiographic scans, which may have led to a different interpretation of echocardiographic images (*i.e*. a small endocarditis lesion may have been picked up by one cardiologist but not another and vice‐versa). In addition, some rhythm abnormalities may have been missed depending on how long the ECG was run for. Furthermore, although the handling and analysis of the aseptic blood cultures were largely similar between each centre, a standardised protocol was not used which could cause some variation in the results. Another limitation was the lack of blood cultures that were positive and the small number of *Bartonella* spp. PCR assays performed. Unfortunately in this study, there were not enough data to allow the analysis of survival between different microorganism causing IE infections. Furthermore, data on routine complete blood work (haematology, biochemistry) were not analysed as part of this study but may have provided useful information to readers. In some cases, the cause of euthanasia may have been due to clients' financial concerns which may not reflect the actual outcome of IE. Unfortunately, this was unlikely to have been written in the clinical notes and must be considered when studying the outcome of the disease.

The results of this study have shown that the bacteria causing this disease are largely similar to those in USA studies, apart from the lack of *Bartonella* spp. and the higher prevalence of mitral compared to aortic valve endocarditis. The number of cases in this study highlights the low frequency of IE out of the total referral population. This study has shown that the mitral valve and large breed dogs appear predisposed to IE which can be caused by a variety of bacteria. Although the prognosis for the disease remains poor, once patients survive to discharge, they can survive for prolonged periods.

### Author contributions

**Margot Berrezaie:** Conceptualization (supporting); formal analysis (lead); investigation (lead); methodology (lead); project administration (lead); visualization (lead); writing – original draft (lead). **David Connolly:** Writing – review and editing (equal). **Judith Cruzado:** Investigation (supporting). **Elzbieta Mederska:** Investigation (supporting). **Joanna Dukes‐McEwan:** Writing – review and editing (equal). **Karen Humm:** Conceptualization (lead); formal analysis (supporting); methodology (supporting); writing – review and editing (equal).

### Conflict of interest

None of the authors of this article has a financial or personal relationship with other people or organisations that could inappropriately influence or bias the content of the paper.
